# Early Estimates of Seasonal Influenza Vaccine Effectiveness — United States, January 2013

**Published:** 2013-01-18

**Authors:** Lisa Jackson, Michael L. Jackson, C. Hallie Phillips, Joyce Benoit, Edward A. Belongia, Deanna Cole, Sarah Kopitzke, Tamara A. Kronenwetter Koepel, Huong Q. McLean, Jennifer K. Meece, Sandra K. Strey, Maria E. Sundaram, Mary Vandermause, Manjusha Gaglani, Juhee Song, Lydia Clipper, Dean Kjar, Anne Robertson, Kempapura Murthy, Melinda Dunnahoo, Stephanie Oliver, Monica Weir, Hope Gonzales, Martha Zayed, Michael Reis, Cathleen Rivera, David Morgan, Pedro Piedra, Vasanthi Avadhanula, Arnold S. Monto, Suzanne E. Ohmit, Joshua G. Petrie, Emileigh Johnson, Rachel T. Cross, Casey Martens, Marcus Zervos, Lois Lamerato, Heather Eng, Mary Patricia Nowalk, Stephen R. Wisniewski, Richard K. Zimmerman, Charles R. Rinaldo, Arlene Bullotta, Joe Suyama, Evelyn Reis, Donald B. Middleton, Rachel Hess, Mark Thompson, Alicia M. Fry, Swathi N. Thaker, Sarah Spencer, Po-Yung Cheng, David K. Shay, LaShondra Berman, Joseph Bresee, Erin Burns, Jerome Tokars, Nancy Cox

**Affiliations:** Group Health Research Institute, Seattle, Washington; Marshfield Clinic Research Foundation, Marshfield, Wisconsin; Scott and White Healthcare, Temple, and Baylor College of Medicine, Houston, Texas; Univ of Michigan, Ann Arbor, and Henry Ford Health System, Detroit; Univ of Pittsburgh Schools of the Health Sciences and UPMC, Pittsburgh, Pennsylvania; Influenza Div, National Center for Immunization and Respiratory Diseases, CDC

In the United States, annual vaccination against seasonal influenza is recommended for all persons aged ≥6 months (1). Each season since 2004–05, CDC has estimated the effectiveness of seasonal influenza vaccine to prevent influenza-associated, medically attended acute respiratory infection (ARI). This season, early data from 1,155 children and adults with ARI enrolled during December 3, 2012–January 2, 2013 were used to estimate the overall effectiveness of seasonal influenza vaccine for preventing laboratory-confirmed influenza virus infection associated with medically attended ARI. After adjustment for study site, but not for other factors, the estimated vaccine effectiveness (VE) was 62% (95% confidence intervals [CIs] = 51%–71%). This interim estimate indicates moderate effectiveness, and is similar to a summary VE estimate from a meta-analysis of randomized controlled clinical trial data (2); final estimates likely will differ slightly. As of January 11, 2013, 24 states and New York City were reporting high levels of influenza-like illness, 16 states were reporting moderate levels, five states were reporting low levels, and one state was reporting minimal levels (3). CDC and the Advisory Committee on Immunization Practices routinely recommend that annual influenza vaccination efforts continue as long as influenza viruses are circulating (1). Persons aged ≥6 months who have not yet been vaccinated this season should be vaccinated. However, these early VE estimates underscore that some vaccinated persons will become infected with influenza; therefore, antiviral medications should be used as recommended for treatment in patients, regardless of vaccination status. In addition, these results highlight the importance of continued efforts to develop more effective vaccines.

To make these interim 2012–13 VE estimates, prospective enrollment of patients at any of the outpatient medical facilities affiliated with the five study sites of the U.S. Influenza Vaccine Effectiveness (Flu VE) Network* began after at least 2 consecutive weeks of laboratory-confirmed cases of influenza were identified through local surveillance.† Details of methods used by the U.S. Flu VE Network have been published previously (4). Trained study staff members reviewed appointment schedules and lists of clinical symptoms and complaints to identify patients with ARI and approached potentially eligible patients (or parents/guardians) to complete a brief screening survey. Patients were eligible for enrollment if they 1) were aged ≥6 months on September 1, 2012, and thus were eligible for vaccination; 2) reported an ARI with onset ≤7 days earlier; and 3) had not been treated with influenza antiviral medication (e.g., oseltamivir). Consenting participants completed an enrollment interview. Because date and type of vaccination were not available for this early estimate, participants were considered vaccinated if they reported having received at least 1 dose of any 2012–13 influenza vaccine before enrollment. At one study site, vaccine receipt was confirmed by a real-time Internet-based vaccine registry (http://www.recin.org) that captures 95% of all influenza vaccinations in that population (5).

Respiratory specimens were collected from each patient using nasal and oropharyngeal swabs, which were placed together in a single cryovial with viral transport medium. Only nasal swabs were collected for patients aged <2 years. Specimens were tested at U.S. Flu VE Network laboratories using CDC’s real-time reverse transcription–polymerase chain reaction (rRT-PCR) protocol for detection and identification of influenza viruses using dual-labeled probe chemistry. VE was estimated as 100% X (1 – odds ratio) using logistic regression, adjusting for study site (4). Stratified analyses were performed by influenza virus type.

Of the 1,155 children and adults with ARI enrolled during December 3, 2012–January 2, 2013, at the five study sites, 416 (36%) tested positive for influenza A or B virus by rRT-PCR; 236 (57%) of these were influenza A, and 180 (43%) were influenza B viruses (Table 1); among 158 subtyped influenza A viruses, all (100%) were influenza A (H3N2) viruses. The 2012–13 seasonal influenza vaccination rate among influenza cases was 32%, compared with 56% among influenza-negative controls. The overall VE (adjusted for study site) for all ages against influenza A and B virus infection associated with medically attended ARI was 62% (CI = 51%–71%) (Table 2). The vaccination rate was lower among influenza B cases (26%) than influenza A cases (37%). The stratified VE against influenza A was 55% (CI = 39%–67%) and against influenza B was 70% (CI = 56%–80%).

## Editorial Note

The early onset of the 2012–13 influenza season offered an opportunity to provide an early VE estimate. Overall, the estimate suggests that the 2012–13 influenza vaccine has moderate effectiveness against circulating influenza viruses, similar to a summary VE estimate from a meta-analysis of randomized controlled clinical trial data (2). Influenza vaccination, even with moderate effectiveness, has been shown to reduce illness, antibiotic use, doctor visits, time lost from work, hospitalizations, and deaths (6). Results for the 2012–13 season indicate that vaccination has reduced the risk for influenza-associated medical visits by approximately 60%, demonstrating the benefits of influenza vaccination during the current season. Influenza activity is likely to continue for several more weeks in the United States. As always, vaccination efforts should continue as long as influenza viruses are circulating. Persons aged ≥6 months who have not yet received the 2012–13 influenza vaccine should be vaccinated. As of January 4, 2013, >128 million doses of influenza vaccine had been distributed in the United States for the 2012–13 season, from approximately 135 million doses that were anticipated to be available for the U.S. market. At this time, some vaccine providers might have exhausted their vaccine supplies. Persons seeking vaccination might need to call more than one provider to locate vaccine.§

What is already known on this topic?

In the United States, annual vaccination against seasonal influenza is recommended for all persons aged ≥6 months. An overall moderate effectiveness for influenza vaccines of approximately 60% has been estimated from a summary of randomized clinical trials. Influenza vaccination, even with moderate effectiveness, can reduce illness, antibiotic use, doctor visits, time lost from work, hospitalizations, and deaths.

What is added by this report?

Based on data from 1,155 children and adults with acute respiratory illness enrolled during December 3, 2012–January 2, 2013, at five study sites with outpatient medical facilities in the United States, the overall estimated effectiveness of the 2012–13 seasonal influenza vaccine for preventing medically attended, laboratory-confirmed influenza virus infection was 62%.

What are the implications for public health practice?

Interim VE estimates indicate the 2012–13 influenza vaccine has moderate effectiveness against circulating influenza viruses, similar to a summary estimate from randomized clinical trials. Vaccination efforts should continue as long as influenza viruses are circulating. Any persons aged ≥6 months who have not received vaccination this season should be vaccinated. However, some vaccinated persons will become infected with influenza. Therefore, antiviral medication should be used as recommended for treatment in patients regardless of their vaccination status.

These early estimates indicate that some vaccinated persons will become infected with influenza, despite having been vaccinated. Therefore, antiviral medications should be used as recommended for treatment in patients regardless of their vaccination status (7).** Antiviral treatment can reduce the duration of illness and complications associated with influenza. Early antiviral treatment is recommended for persons with suspected influenza with severe or progressive illness (e.g., hospitalized persons) and those at high risk for complications from influenza, no matter how severe the illness. Antiviral treatment should be started as early as possible, preferably within 48 hours after illness onset. Among hospitalized patients, however, treatment should be initiated on admission; several studies suggest that antiviral treatment reduces mortality and illness severity among hospitalized adults, even when initiated ≥48 hours after illness onset (7). The decision to initiate antiviral treatment should not wait for laboratory confirmation of influenza and should not be dependent on insensitive assays, such as rapid influenza diagnostic tests.

Although these early VE estimates differ for influenza A and influenza B virus infection, the CIs overlap, and the actual difference might be less. Early observations from the U.S. Flu VE Network are consistent with data from national domestic surveillance, which indicates that most influenza A viruses circulating in the United States thus far are influenza A (H3N2) viruses (3). Of the subset of influenza A (H3N2) viruses and B viruses characterized at CDC this season, the majority are antigenically like the influenza A (H3N2) and B component of the 2012–13 seasonal vaccine (3).

The findings in this report are subject to at least four limitations. First, VE can differ for patients of different ages, and age data were not yet available from all sites. However, VE estimates from one site (Wisconsin) differed little before (69%) and after age-adjustment (66%) (Edward Belongia, Marshfield Clinic Research Foundation, personal communication, January 2013). Second, vaccination status was self-reported; dates of vaccination were not available, except from one site; and vaccine formulation was not known. However, experience from prior seasons suggests that few persons are vaccinated <2 weeks from illness onset (4), a period when vaccine might not be effective yet, and self-reported influenza vaccine status was sensitive and fairly specific compared with documented vaccination at an immunization registry (5). Vaccination dates will be available for subsequent VE estimates. Third, VE estimates for prior seasons were reduced after adjusting for potential confounding factors (4), and the fully adjusted VE estimate for this season likely will be lower, also. Observational VE studies, such as those used for the current estimates, have greater potential for confounding and bias relative to randomized clinical trials, particularly when diagnostic test specificity is low (8). However, the U.S. Flu VE Network study design attempts to minimize bias and confounding through systematic screening of eligible patients and use of a highly sensitive and specific endpoint (rRT-PCR–confirmed influenza). Finally, subsequent VE estimates might change during the season if circulating viruses or population immunity change over the course of the season.

CDC will monitor VE throughout the season and provide updates. Although influenza vaccines are the best tool for prevention of influenza currently available, more effective vaccines are needed. Antiviral medications continue to be an important adjunct in the treatment and control of influenza and should be used as recommended, regardless of patient vaccination status.

## Figures and Tables

**TABLE 1:** Number and percentage of outpatients aged ≥6 months with acute respiratory illness, by influenza virus test result and study site — U.S. Influenza Vaccine Effectiveness Network,* United States, December 3, 2012–January 2, 2013

	Patients by influenza test result							
		
	Influenza A viruses		Influenza B viruses		Influenza-negative		Total patients	
				
Location	No.	(%)	No.	(%)	No.	(%)	No.	(%)
Washington	8	(21)	1	(3)	29	(76)	**38**	**(3)**
Wisconsin	77	(20)	127	(32)	190	(48)	**394**	**(34)**
Michigan	18	(19)	8	(8)	70	(73)	**96**	**(8)**
Pennsylvania	83	(33)	0	—	168	(67)	**251**	**(22)**
Texas	50	(13)	44	(12)	282	(75)	**376**	**(33)**
**All sites**	**236**	**(20)**	**180**	**(16)**	**739**	**(64)**	**1,155**	**(100)**

* The U.S. Flu VE Network includes Group Health Cooperative (Seattle, Washington), the Marshfield Clinic Research Foundation (Marshfield, Wisconsin), the University of Michigan School of Public Health (the University of Michigan School of Public Health, partnered with the University of Michigan Health System, Ann Arbor, and the Henry Ford Health System, Detroit), the University of Pittsburgh Schools of the Health Sciences (the University of Pittsburgh Schools of the Health Sciences, partnered with UPMC, Pittsburgh, Pennsylvania), and Scott and White Healthcare (Temple, Texas).

**TABLE 2:** Number and percentage of persons vaccinated with 2012–13 seasonal trivalent influenza vaccine among influenza-positive case-patients and influenza-negative controls, and vaccine effectiveness* against all influenza viruses and influenza virus types A and B among 1,155 outpatients with acute respiratory illness — U.S. Influenza Vaccine Effectiveness Network,† United States, December 3, 2012–January 2, 2013

	Influenza-positive cases		Influenza-negative controls		Vaccine effectiveness	
			
Virus	No. vaccinated/Total	(%)	No. vaccinated/Total	(%)	(%)	(95% CI)
Influenza A and B	133/**416**	(32)	411/**739**	(56)	(62)	(51–71)
Influenza A only	87/**236**	(37)	411/**739**	(56)	(55)	(39–67)
Influenza B only	46/**180**	(26)	411/**739**	(56)	(70)	(56–80)

**Abbreviation:** CI = confidence interval.

* Adjusted for study site.

† The U.S. Flu VE Network includes Group Health Cooperative (Seattle, Washington), the Marshfield Clinic Research Foundation (Marshfield, Wisconsin), the University of Michigan School of Public Health (the University of Michigan School of Public Health, partnered with the University of Michigan Health System, Ann Arbor, and the Henry Ford Health System, Detroit), the University of Pittsburgh Schools of the Health Sciences (the University of Pittsburgh Schools of the Health Sciences, partnered with UPMC, Pittsburgh, Pennsylvania), and Scott and White Healthcare (Temple, Texas).

## Footnotes

* The U.S. Flu VE Network includes Group Health Cooperative (Seattle, Washington), the Marshfield Clinic Research Foundation (Marshfield, Wisconsin), the University of Michigan School of Public Health (the University of Michigan School of Public Health, partnered with the University of Michigan Health System, Ann Arbor, and the Henry Ford Health System, Detroit), the University of Pittsburgh Schools of the Health Sciences (the University of Pittsburgh Schools of the Health Sciences, partnered with UPMC, Pittsburgh, Pennsylvania), and Scott and White Healthcare (Temple, Texas).

† Beginning enrollment dates were December 3, 2012, in Pittsburgh; December 9, 2012, in Temple; December 17, 2012, in Marshfield and Ann Arbor; December 26, 2012, in Seattle; and January 2, 2013, in Detroit.

§ An influenza vaccine locator is available at http://flushot.healthmap.org/?address.

** A complete summary of guidance for antiviral use is available at http://www.cdc.gov/flu/professionals/antivirals/index.htm.
